# Waist-to-height ratio and non-alcoholic fatty liver disease in adults

**DOI:** 10.1186/s12876-021-01824-3

**Published:** 2021-05-25

**Authors:** Guotai Sheng, Qiyang Xie, Rongsheng Wang, Chong Hu, Mingchun Zhong, Yang Zou

**Affiliations:** 1 Cardiology Department, Jiangxi Provincial People’s Hospital Affiliated to Nanchang University, Nanchang, 330006 Jiangxi Province China; 2 Department of Intensive Care Unit, Jiangxi Provincial People’s Hospital Affiliated to Nanchang University, Nanchang, 330006 Jiangxi Province China; 3 Gastroenterology Department, Jiangxi Provincial People’s Hospital Affiliated to Nanchang University, Nanchang, 330006 Jiangxi Province China

**Keywords:** Waist-to-height ratio, Adults, Saturation effects, Threshold effects, Non-linear, Non-alcoholic fatty liver disease

## Abstract

**Background:**

The waist-to-height ratio (WHtR) has been recognised as a powerful indicator to evaluate non-alcoholic fatty liver disease (NAFLD) in recent years, but few related studies are available. Thus, clarifying the association between the WHtR and NAFLD may be beneficial to the prevention and treatment of NAFLD.

**Methods:**

The cross-sectional study population was from a large-scale health examination programme called ‘human dock’ in Japan. In this study, 14,125 participants in this health examination programme were included. To understand the association between the WHtR and NAFLD more intuitively, we grouped the WHtR values into quintiles and used a multivariable logistic regression model to assess WHtR and its quintile with NAFLD risk. Moreover, we used the generalised additive model to model the association between WHtR and NAFLD to explore their non-linear relationship.

**Results:**

The prevalence of NAFLD among participants in this study was 17.59%, with an average age of 43.53 ± 8.89 years. After adjusting for all non-collinear covariables, we observed a 66% increase in the NAFLD risk per SD increase in WHtR. Furthermore, in the quintile groups of WHtR, the participants in quintile 2, quintile 3, quintile 4, and quintile 5 had 3.62-fold, 5.98-fold, 9.55-fold, and 11.08-fold increased risks of NAFLD, respectively, compared with those in quintile 1 (*P*_trend_ < 0.0001). Non-linear relationship analysis revealed threshold and saturation effects between WHtR and NAFLD in which a WHtR of approximately 0.4 might be the threshold effect of NAFLD risk, 0.6 might be the saturation effect of NAFLD risk. Additionally, subgroup analysis showed that the interaction between WHtR and BMI was significant.

**Conclusions:**

Our results suggest that in adults, the WHtR is associated with NAFLD, and the association is not purely linear but non-linear, with significant threshold and saturation effects.

**Supplementary Information:**

The online version contains supplementary material available at 10.1186/s12876-021-01824-3.

## Background

In recent decades, with the rapid development of the social economy and change in lifestyle, the prevalence of non-alcoholic fatty liver disease (NAFLD) has risen sharply worldwide. Estimates suggest that more than 1/4 of individuals worldwide have NAFLD, which has become one of the most common chronic diseases [1–3]. Similar to the characteristics of other chronic diseases, NAFLD usually has a long course of illness, affects multiple organ systems of the body, and has many complications and low public awareness rates [1, 4–6]. However, because the physical injuries caused by NAFLD are often chronic and asymptomatic, the actual prevalence rate may be underestimated, creating a vast hidden burden of disease to the already overburdened health care system [2–4, 6, 7]. Combined with the previous valuable experience in exploring the pathogenic features of hypertension, diabetes, and other chronic diseases in humans [8–10], we believe that preventing the occurrence and development of NAFLD will be the primary goal in the future. Thus, the early detection of important risk factors for NAFLD is the most important task presently [7].

The waist circumference (WC) and body mass index (BMI) are currently the most widely used anthropometric indicators to assess obesity worldwide and are also the most critical risk factors for NAFLD [1, 3, 11]. These indicators are simple to measure and provide excellent convenience to prevent and manage many diseases [12, 13]. However, in recent years, in-depth studies have found that the waist-to-height ratio (WHtR) can better assess the risk of central obesity, diabetes, hypertension, and other metabolic diseases [14–17]. Furthermore, several recent cross-sectional studies have shown that WHtR is a stronger predictor of NAFLD risk and its severity and is more sensitive to diagnosis than WC and BMI [18, 19]. However, few studies have investigated the association between WHtR and NAFLD, which contain deeper relationships, such as non-linear relationships, and whether a special population exists among different subgroups. Additionally, the sample sizes of several existing studies on the association between WHtR and NAFLD are relatively small (N = 250–6143). Therefore, this study aimed to further explore and analyse the association between WHtR and NAFLD in adults using a large sample size.

## Methods

### Subject population and design

Our study population was from a large-scale health examination programme called ‘human dock’ in Japan that aims to promote public health and assesses common chronic diseases and their risk factors through physical examination. The research data have been uploaded to Dryad public database by Okamura et al. According to the terms of service of Drayad database, we can use this data for secondary data analysis based on different assumptions [20]. In previous studies, research ethics was approved by the Murakami Memorial Hospital Ethics Committee and informed consent was obtained from all subjects; Additionally, in order to protect the privacy of patients, the effective identification ID of all subjects in the study was replaced by health codes, and the whole study process followed the Declaration of Helsinki. Since this study was a secondary analysis of previous studies, there was no need to apply for separate ethical approval.

In previous studies, the researchers used a longitudinal design and analysed the risk factors for diabetes [20]. In this study, we adopted a cross-sectional design that included participants in the ‘human dock’ physical examination programme from 2004 to 2015. We have added some exclusion criteria to the previous research design: (a) male alcohol consumption ≥ 210 g/w and female alcohol consumption ≥ 140 g/w [21]; (b) viral hepatitis or diabetes diagnosed at baseline; (c) on medication at baseline; (d) impaired fasting blood glucose (FPG); (e) age < 18 years and (e) missing covariant data.

### Data collection

As mentioned previously [20], the clinical baseline information in this study was collected through a standardised self-administered questionnaire, including age, sex, height, weight, WC, smoking status, drinking status, systolic blood pressure (SBP), diastolic blood pressure (DBP) and habit of exercise. Information such as height, weight and WC came from the patient's self-report. The habit of exercise was defined as participating in any exercise more than once a week; Smoking status was divided into nonsmokers, former smokers, and current smokers by asking about smoking history when baseline data were collected. Drinking status was divided into non-drinking or small drinking (< 40 g/w), light drinking (40–139 g/w) and moderate drinking (140–209 g/w) depending on the amount of alcohol consumed. BMI was calculated as weight/height^2^, and WHtR as WC/height. Haematological indicators were tested by sampling venous blood after a night of fasting and included gamma-glutamyl transferase (GGT), alanine aminotransferase (ALT), high-density lipoprotein cholesterol (HDL-C), aspartate aminotransferase (AST), total cholesterol (TC), haemoglobin A1c (HBA1c), triglyceride (TG), and FPG.

### Diagnosis of NAFLD

NAFLD was assessed by abdominal ultrasound, and experienced gastroenterologists reviewed the ultrasound images without knowing the participants’ personal information. According to the results of four types of ultrasound, such as liver brightness, hepatorenal echo contrast, vascular blurring, and deep attenuation, the evaluation was made and a final diagnosis was made [22].

### Statistical analysis

All the analyses in this study were performed using R (version 3.4.3) and Empower (R) (version 2.0) statistical software. To understand the association between WHtR and other variables more intuitively, we grouped WHtR into quintiles, and the linear trend of baseline characteristics was tested by logical regression or linear regression. Additionally, we used a multivariate logical regression model to examine the NAFLD risk corresponding to each WHtR quintile. As a sensitivity analysis, we treated the WHtR quintile as a continuous variable and examined the WHtR quintile and NAFLD risk trend. In multivariate analysis, the adjustment rules of variables were followed according to the Strengthening the Reporting of Observational Studies in Epidemiology (STROBE) statement [23]. The adjustment results of different degrees were shown in different models, where model 1 was the unadjusted model, model 2 adjusted the basic demographic variables (adjusted for age and sex), model 3 adjusted for variables whose impact on the WHtR and NAFLD matching risk was greater than 10% (adjusted for BMI, ALT, HDL-C, TG and height) [24], and model 4 adjusts all non-collinear variables (collinearity diagnosis discriminates according to variance inflation factor [25], Additional file 1: Table S1). We also checked whether a non-linear relationship existed between WHtR and NAFLD using the generalised additive model (GAM, spline smoothing function). Finally, to further explore the association between WHtR and NAFLD, we used the likelihood ratio test to examine the interaction between the pre-determined subgroup (sex, age, BMI, habit of exercise, and drinking status) and WHtR.

In this study, the distribution pattern of the quantitative variables was judged by the QQ plot, and the expression was summarised as medians (quartile interval) or means ± standard deviation (SD). The frequency of qualitative variables was reported as a percentage, and chi-squared test evaluated the differences between groups.

## Results

### Baseline characteristics of WHtR

In total, 20,944 participants were enrolled, among which 6693 participants were excluded because they did not meet the screening criteria (736 participants with viral hepatitis or diabetes, 2321 participants were on medication at baseline, 808 participants had impaired FPG, 1952 participants were heavy drinkers, 10 participants for unknown reasons and 863 participants with missing covariates), leaving 14,251 adults for analysis, including 7411 male and 6840 female, with an average age of 43.53 ± 8.89 years.

In this study, the WHtR values ranged from 0.28 to 0.79, and Table 1 evaluates the association between the baseline data and WHtR quintiles. With the gradual increase in WHtR, the proportion of male participants gradually increased, the age, height, weight, BMI, WC, SBP, and DBP of all participants showed a gradual increase, the NAFLD patients, smokers and drinkers, and the levels of ALT, AST, GGT, TC, TG, HbA1c and FPG were also on the increased (all *P*_trend_ < 0.05). Notably, when the WHtR was elevated, we found that the number of individuals who maintained the habit of exercise gradually decreased, and the HDL-C levels were also decreased (*P*_trend_ < 0.05).

**Table 1:** Baseline characteristics of five groups

	WHtR quintile					*P*-trend
	Q1(≥ 0.28, ≤ 0.42)	Q2(> 0.42, ≤ 0.45)	Q3(> 0.45 ≤ 0.47)	Q4(> 0.47, ≤ 0.50)	Q5 (> 0.50, ≤ 0.79)	
No. of participants	2850	2849	2851	2848	2853	
Age	39 (35–45)	40 (36–47)	42 (37–49)	44 (38–52)	46.00 (40–54)	< 0.01
Sex (male)	962 (33.75%)	1313 (46.09%)	1624 (56.96%)	1792 (62.92%)	1720 (60.29%)	< 0.01
NAFLD	12 (0.42%)	97 (3.40%)	312 (10.94%)	731 (25.67%)	1355 (47.49%)	< 0.01
Habit of exercise	522 (18.32%)	555 (19.48%)	511 (17.92%)	480 (16.85%)	402 (14.09%)	< 0.01
BMI (kg/m^2^)	18.76 ± 1.47	20.47 ± 1.46	21.77 ± 1.60	23.27 ± 1.76	26.05 ± 2.90	< 0.01
Height (cm)	164.03 ± 8.31	164.69 ± 8.51	165.50 ± 8.46	165.70 ± 8.38	164.07 ± 8.61	0.03
Weight (kg)	49.7 (45.4–55.5)	55.2 (49.4–61.6)	60.00 (53.2–66.4)	64.40 (56.9–71.1)	69.60 (61.8–78.4)	< 0.01
WC (cm)	65.28 ± 4.28	71.26 ± 3.97	75.91 ± 4.06	80.53 ± 4.33	87.93 ± 6.68	< 0.01
ALT (IU/L)	14 (11–18)	15 (12–19)	17.00 (12–22)	18 (14–25)	22 (16–32)	< 0.01
AST (IU/L)	16 (13–19)	16 (13–20)	17.00 (14–20)	18 (14–21)	19 (16–24)	< 0.01
GGT (IU/L)	12.00 (10–15)	13 (10–17)	15 (11–21)	17 (12–24)	20 (14–29)	< 0.01
HDL-C (mmol/L)	1.67 ± 0.40	1.56 ± 0.38	1.45 ± 0.39	1.35 ± 0.37	1.27 ± 0.34	< 0.01
TC (mmol/L)	4.85 ± 0.82	4.95 ± 0.81	5.12 ± 0.84	5.24 ± 0.86	5.45 ± 0.88	< 0.01
TG (mmol/L)	0.53 (0.38–0.71)	0.60 (0.43–0.86)	0.73 (0.50–1.05)	0.88 (0.61–1.32)	1.06 (0.71–1.55)	< 0.01
HbA1c (%)	5.10 (4.90–5.30)	5.10 (4.90–5.40)	5.15 (5.00–5.40)	5.20 (5.00–5.40)	5.25 (5.10–5.50)	< 0.01
FPG (mmol/L)	4.97 ± 0.40	5.05 ± 0.39	5.17 ± 0.39	5.24 ± 0.40	5.32 ± 0.38	< 0.01
SBP (mmHg)	106.16 ± 12.56)	109.57 ± 12.55	113.72 ± 13.49	117.05 ± 13.64	123.16 ± 15.52	< 0.01
DBP (mmHg)	65.89 ± 8.59	67.91 ± 8.91	70.94 ± 9.64	73.45 ± 9.88	77.41 ± 10.63	< 0.01
Smoking status						< 0.01
Non	2081 (73.02%)	1848 (64.86%)	1681 (58.96%)	1530 (53.72%)	1606 (56.29%)	
Former	298 (10.46%)	444 (15.58%)	558 (19.57%)	658 (23.10%)	601 (21.07%)	
Current	471 (16.53%)	557 (19.55%)	612 (21.47%)	660 (23.17%)	646 (22.64%)	
Drinking status						< 0.01
Non or small (< 40 g/w)	2522 (88.49%)	2399 (84.20%)	2285 (80.15%)	2256 (79.21%)	2343 (82.12%	
Light (40–139 g/w)	264 (9.26%)	338 (11.86%)	404 (14.17%)	402 (14.12%)	350 (12.27%)	
Moderate (140–209 g/w)	64 (2.25%)	112 (3.93%)	162 (5.68%)	190 (6.67%)	160 (5.61%)	

Values were expressed as mean ± SD or n (%)

*NAFLD* nonalcoholic fatty liver disease, *BMI* body mass index, *WC* waist circumference, *ALT* alanine aminotransferase, *AST* aspartate aminotransferase, *GGT* gamma-glutamyl transferase, *HDL-C* high-density lipoprotein cholesterol, *TC* total cholesterol, *TG* triglyceride, *HbA1c* hemoglobin A1c, *FPG* fasting plasma glucose, *SBP* systolic blood pressure, *DBP* Diastolic blood pressure

### Baseline characteristics of NAFLD

Table 2 shows the baseline characteristics of the participants diagnosed with NAFLD and non-NAFLD. Among the 14,251 participants, the prevalence of NAFLD was 17.59%, mainly male patients (80.93%). In the NAFLD group, the general clinical indexes (age, height, weight, BMI, WC, WHtR, SBP, DBP, smoking status, and drinking status) of the participants were higher than those in the non-NAFLD group (*P* < 0.001). Additionally, among the participants with confirmed NAFLD, significantly fewer participants had a habit of exercise (15.04%). Furthermore, except for HDL-C, the haematological indicators of the NAFLD participants were all higher than those of the non-NAFLD participants (*P* < 0.001).

**Table 2:** Baseline characteristics of the NAFLD and non-NAFLD groups

	Non-NAFLD	NAFLD	*P*-value
No. of participants	11,744	2507	
Age (years)	42.00 (36.00–49.00)	44.00 (38.00–51.00)	< 0.01
Sex (male)	5382 (45.83%)	2029 (80.93%)	< 0.01
Habit of exercise (n%)	2093 (17.82%)	377 (15.04%)	< 0.01
BMI (kg/m^2^)	21.33 ± 2.61	25.50 ± 3.13	< 0.01
Height (cm)	164.11 ± 8.44	168.03 ± 7.90	< 0.01
Weight (kg)	56.70 (50.00–64.40)	71.20 (64.40–78.70)	< 0.01
WC (cm)	74.00 (68.00–79.50)	85.30 (81.00–90.50)	< 0.01
ALT (IU/L)	15.00 (12.00–20.00)	27.00 (20.00–39.00)	< 0.01
AST (IU/L)	17.00 (14.00–20.00)	20.00 (17.00–26.00)	< 0.01
GGT (IU/L)	14.00 (11.00–18.00)	23.00 (16.00–33.00)	< 0.01
HDL-C (mmol/L)	1.52 ± 0.40	1.18 ± 0.29	< 0.01
TC (mmol/L)	5.06 ± 0.85	5.44 ± 0.87	< 0.01
TG (mmol/L)	0.65 (0.45–0.95)	1.24 (0.87–1.80)	< 0.01
HbA1c (%)	5.10 (4.90–5.40)	5.30 (5.10–5.50)	< 0.01
FPG (mmol/L)	5.09 ± 0.40	5.39 ± 0.36	< 0.01
SBP (mmHg)	111.91 ± 14.02	123.41 ± 14.83	< 0.01
DBP (mmHg)	69.69 ± 9.85	77.81 ± 10.19	< 0.01
WHtR	0.45 ± 0.04	0.51 ± 0.05	< 0.01
Smoking status			0.16
Non	7561 (64.38%)	1185 (47.27%)	
Former	1920 (16.35%)	639 (25.49%)	
Current	2263 (19.27%)	683 (27.24%)	
Drinking status		/	< 0.01
Non or small (< 40 g/w)	9717 (82.74%)	2088 (83.29%)	
Light (40–139 g/w)	1472 (12.53%)	286 (11.41%)	
Moderate (140–209 g/w)	555 (4.73%)	133 (5.31%)	

Abbreviations as in Table 1

### Association between WHtR and NAFLD

In the multivariate logistic regression model, WHtR was significantly positively correlated with NAFLD, and this association remained unchanged in the unadjusted model (model 1), the basic adjusted model (model 2), the model adjusted according to the method recommended by the STROBE statement (model 3), and the model adjusted for all non-collinear variables (model 4). In the model that adjusted all the non-collinear variables, we observed a 66% increase in NAFLD risk for per SD increase in WHtR (adjusted odds ratio (OR):1.66; 95% confidence interval (CI): 1.45, 1.89). Additionally, among the quintile groups of WHtR, participants in quintile 2, quintile 3, quintile 4, and quintile 5 showed 3.62-fold, 5.98-fold, 9.55-fold, and 11.08-fold increased risks of NAFLD, respectively, compared with that in quintile 1 (*P* trend < 0.0001) (Table 3). These results suggest that individuals with higher WHtR are more likely to develop NAFLD than those with lower WHtR.

**Table 3:** Logistic regression analyses for the relationship between WHtR and incident NAFLD in different models

	Model 1	Adjusted odds ratios (95% confidence interval)		
		Model 2*	Model 3^&^	Model 4^#^
WHtR (per SD increase)	4.06 (3.82, 4.31)	4.49 (4.20, 4.80)	1.84 (1.64, 2.08)	1.66 (1.45, 1.89)
WHtR (quintile)				
Quintile 1	Ref	Ref	Ref	Ref
Quintile 2	8.34 (4.57, 15.22)	7.22 (3.95, 13.19)	4.11 (2.23, 7.57)	3.62 (1.92, 6.68)
Quintile 3	29.06 (16.29, 51.86)	22.98 (12.86, 41.08)	7.47 (4.12, 13.53)	5.98 (3.30, 10.85)
Quintile 4	81.66 (46.04, 144.87)	65.10 (36.62, 115.74)	12.42 (6.84, 22.53)	9.55 (5.24, 17.40)
Quintile 5	213.92 (120.77, 378.93)	202.18 (113.80, 359.18)	15.42 (8.33, 28.53)	11.08 (5.94, 20.68)
*P*-trend	< 0.01	< 0.01	< 0.01	< 0.01

Model 1 was the unadjusted model

^*^Adjusted for sex and age

^&^Adjusted for BMI, ALT, HDL-C, TG and height

^#^Adjusted for sex, age, BMI, ALT, AST, habit of exercise, GGT, HDL-C, TC, TG, HbA1C, smoking status, FPG, SBP, height and drinking status

**Fig. 1:**
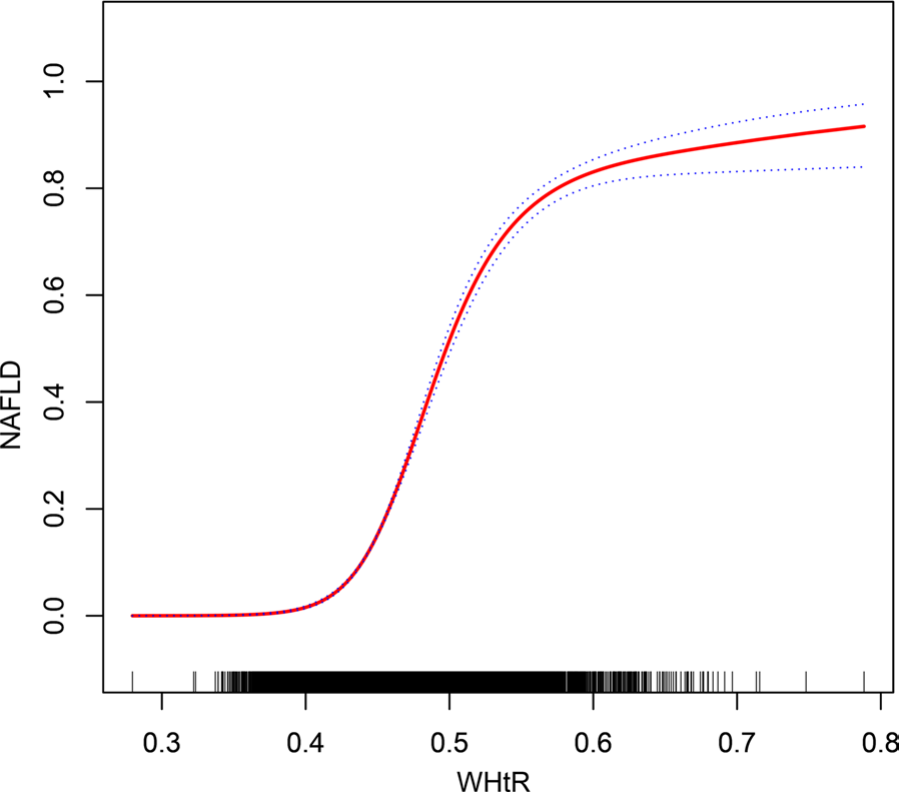
Non-linear association between the WHtR and NAFLD in adults. The dashed lines are 95% confidence intervals

We continued to use GAM to model the association between WHtR and NAFLD. The regression spline curve indicated that the relationship between WHtR and NAFLD was non-linear, in which a WHtR of approximately 0.4 might be the threshold effect of NAFLD risk, 0.6 might be the saturation effect of NAFLD risk, and a value between 0.4 and 0.6 might demonstrate linear relationship with the risk of NAFLD (Fig. 1).

### Subgroup analysis

To further reveal the deep association between WHtR and NAFLD, interaction tests were performed in the pre-defined subgroups (Table 4). Among these subgroups, we found a significant interaction between WHtR and BMI (*P* _interaction_ < 0.01). Among them, this risk association was significantly higher in non-obese individuals than in those with BMI ≥ 24 kg/m^2^ (adjusted OR: 2.73 vs 1.94). Additionally, the interaction tests for age, sex, habit of exercise, and drinking status were not significant (*P*_interaction_ > 0.05).

**Table 4:** Stratified associations between WHtR and NAFLD by age, sex, habit of exercise, BMI, and drinking status

Subgroup	No. of participants	No. of cases	Adjusted OR (95%CI)	*P*interaction
Age (years)				0.31
18–29	401	22 (0.88%)	1.87 (0.99, 3.55)	
30–39	4885	740 (29.52%)	1.90 (1.58, 2.28)	
40–49	5278	979 (39.05%)	1.57 (1.34, 1.84)	
50–59	3052	659 (26.29%)	1.69 (1.43, 1.99)	
≥ 60	635	107 (4.27%)	1.57 (1.18, 2.09)	
Sex				0.80
Male	7411	2029 (80.93%)	1.64 (1.42, 1.90)	
Female	6840	478 (19.07%)	1.67 (1.44, 1.95)	
Habit of exercise				0.61
Yes	2470	377 (15.04%)	1.73 (1.41, 2.12)	
No	11,781	2130 (84.96%)	1.64 (1.43, 1.88)	
BMI (kg/m^2^)				< 0.01
≤ 18.5	1545	3 (0.12%)	2.93 (0.35, 24.24)	
≥ 18.5, < 24	9336	847 (33.79%)	2.73 (2.37, 3.14)	
≥ 24	3370	1657 (66.09%)	1.94 (1.72, 2.18)	
Drinking status				0.73
Non or small (< 40 g/w)	11,805	2088 (83.29%)	1.70 (1.48, 1.94)	
Light (40–140 g/w)	1758	286 (11.41%)	1.57 (1.24, 1.98)	
Moderate (140–209 g/w)	688	133 (5.31%)	1.79 (1.26, 2.56)	

Adjusted for sex, age, BMI, ALT, AST, habit of exercise, GGT, HDL-C, TC, TG, HbA1C, smoking status

FPG, SBP, height and drinking status

## Discussion

This cross-sectional study, based on the general adult population, examined the association between the WHtR and the prevalence of NAFLD and found that the risk of NAFLD was significantly associated with an elevated WHtR value. Even after adjusting for all the non-collinear covariates, the association remained strong and positive (adjusted OR: 1.66; 95% CI: 1.45, 1.89), and this risk association was more significant in non-obese individuals (adjusted OR: 2.73; 95% CI: 2.37, 3.14; *P*_interaction_ = 0.0006).

The prevalence of NAFLD in this study was 17.59%, which was relatively low compared with that in Asia (27.37%) [2]. The cause may be related to a significant proportion of participants in this study still maintaining their habit of exercise (17.33%). It is well known that regular exercise can reduce the risk of NAFLD and is currently the primary way to prevent NAFLD occurrence and control NAFLD development [1, 4, 5, 26]. In this study, 84.96% of patients with NAFLD were from individuals who had no habit of exercise (Table 4). Additionally, although the prevalence of NAFLD in non-obese people (BMI < 24 kg/m^2^) was lower than that in overweight or obese people (BMI ≥ 24 kg/m^2^), the subgroup analysis showed that the risk of NAFLD in non-obese people was significantly higher than that in overweight or obese people (adjusted OR: 2.73 vs 1.94, *P*_interaction_ < 0.01). In some recent studies, researchers have expressed similar concerns, believing that non-obese individuals in Asia are vulnerable to NAFLD and the health problems of this unique population should be given attention [2, 18, 27, 28].

Compared with other studies on the association between WHtR and NAFLD [18, 19, 28–30], the main advantage of this study was based on the large sample data, further analysis of various potential confounding factors to perform strict statistical adjustment, revealing the positive association between WHtR and NAFLD, and examining the possible unique population through hierarchical analysis and interactive tests. Additionally, our analysis showed a non-linear relationship between WHtR and NAFLD. A WHtR of approximately 0.4 likely indicated a threshold effect of NAFLD risk; 0.6 might be a saturation effect on the risk of NAFLD. A WHtR value between 0.4 and 0.6 likely indicated a linear relationship between WHtR and NAFLD risk. In previous studies, researchers generally believed that the optimal cut-off value for WHtR was approximately 0.5 [18, 19, 29–31]; our latest evidence also supports this conclusion, and we visualised the association between WHtR and NAFLD. We believe that when WHtR is in the range of 0.4–0.6, the risk of NAFLD must be evaluated carefully. To our best knowledge, this is the first study to demonstrate a non-linear association between WHtR and the risk of NAFLD.

The mechanism by which WHtR correlates with NAFLD remains unclear. However, related studies have shown that WHtR is closely related to chronic inflammation and insulin resistance (IR), which may lead to NAFLD occurrence [32, 33]. As explained in the ‘parallel multiple-hit theory’, IR is the primary factor that triggers NAFLD [34]. IR causes an increase in free fatty acids in hepatocytes, and these fat molecules make the liver more vulnerable to a second blow, increasing the liver’s susceptibility to other damage factors [35, 36]. Additionally, chronic inflammation can cause hepatocyte stress and IR, which lead to lipid accumulation and form a vicious circle, further increasing the risk of NAFLD [37, 38].

### Study strengths and limitations

This study’s advantage is that, based on a large sample size, a standard questionnaire was used to evaluate the habit of exercise as an important risk factor, and a rigorous statistical adjustment was made to explore the association between WHtR and NAFLD deeply. Additionally, this study is the first to confirm that the association between WHtR and NAFLD is non-linear and provides a reference range of WHtR associated with NAFLD risk.

This study’s limitations are mainly due to the following aspects: (a) a cross-sectional design was adopted; thus, the causal association between WHtR and NAFLD could not be determined. However, due to the retrospective nature of the study, the issue of observational bias was avoided. Additionally, the data analysis in this study was based on a large sample, and the conclusions drawn could be considered relatively reliable; (b) IR was not evaluated; thus, regarding the association between WHtR and NAFLD, we can only speculate that the mechanism of IR may be involved in one of the important links; (c) the diagnosis of NAFLD was evaluated by abdominal colour ultrasound, and the true prevalence of NAFLD may be underestimated compared with liver biopsies—that is, the effect of WHtR on NAFLD may also be underestimated. However, abdominal colour ultrasound has reduced the economic burden and physical damage of physical examination in healthy individuals; with the improvement in ultrasonic detection technology, the sensitivity and specificity of abdominal colour ultrasound in detecting NAFLD have reached a high level [39]; (d) although we have adjusted a large range of known risk factors, many risk factors may exist that we have not yet discovered or cannot measure, causing inevitable residual confusion; (e) since this study is based on a secondary analysis of previous research data [20], diabetes patients are not included in the data package, so it may cause a certain selection bias. But from another point of view, the positive correlation between diabetes and NAFLD has been proved many times in many previous studies [1, 2, 6], but this study still found that there is a positive correlation between WHtR and NAFLD even excluding patients with diabetes, so it can be considered that this association is relatively reliable.

## Conclusion

In summary, the results of this study suggest that in adults, the WHtR is positively associated with NAFLD, and the relationship between the two is non-linear, with significant threshold and saturation effects. These findings further enrich the latest evidence and may benefit the public in terms of the prevention and intervention of NAFLD. Given that the WHtR is a repeatable and easy-to-measure indicator, we suggest that WHtR should be promoted as a routine programme to screen adults for NAFLD.

## Supplementary Information

**Additional file 1. Table S1** Collinearity diagnostics steps.

## Data Availability

The datasets were acquired from the DRYAD database [https://datadryad.org].

## Abbreviations

STROBE: Strengthening the Reporting of Observational Studies in Epidemiology
SD: Standard deviation
BMI: Body mass index
SBP: Systolic blood pressure
NAFLD: Non-alcoholic fatty liver disease
TG: Triglyceride
CI: Confidence interval
IR: Insulin resistance
CI: Confidence interval
WHtR: Waist-to-height ratio
AST: Aspartate aminotransferase
FPG: Fasting plasma glucose
WC: Waist circumference
DBP: Diastolic blood pressure
GGT: Gamma-glutamyl transferase
ALT: Alanine aminotransferase
OR: Odds ratios
TG: Triglyceride
GAM: Generalized additive model
TC: Total cholesterol
HDL-C: High-density lipoprotein cholesterol
HBA1c: Hemoglobin A1c

## References

[1] Wang XJ, Malhi H (2018). Nonalcoholic fatty liver disease. Ann Intern Med.

[2] Younossi ZM, Koenig AB, Abdelatif D, Fazel Y, Henry L, Wymer M (2016). Global epidemiology of nonalcoholic fatty liver disease-Meta-analytic assessment of prevalence, incidence, and outcomes. Hepatology.

[3] Bernstein DE (2018). Nonalcoholic fatty liver disease: an expanding health care epidemic. Clin Liver Dis.

[4] Cai J, Zhang XJ, Li H (2019). Progress and challenges in the prevention and control of nonalcoholic fatty liver disease. Med Res Rev.

[5] Lonardo A, Nascimbeni F, Maurantonio M, Marrazzo A, Rinaldi L, Adinolfi LE (2017). Nonalcoholic fatty liver disease: evolving paradigms. World J Gastroenterol.

[6] Byrne CD, Targher G (2015). NAFLD: a multisystem disease. J Hepatol.

[7] Araújo AR, Rosso N, Bedogni G, Tiribelli C, Bellentani S (2018). Global epidemiology of non-alcoholic fatty liver disease/non-alcoholic steatohepatitis: what we need in the future. Liver Int.

[8] Whelton PK, Carey RM, Aronow WS, Casey DE, Collins KJ, Dennison Himmelfarb C (2018). 2017 ACC/AHA/AAPA/ABC/ACPM/AGS/APhA/ASH/ASPC/NMA/PCNA Guideline for the Prevention, Detection, Evaluation, and Management of High Blood Pressure in Adults: a report of the American College of Cardiology/American Heart Association Task Force on Clinical Practice Guidelines. Hypertension.

[9] Xie F, Chan JC, Ma RC (2018). Precision medicine in diabetes prevention, classification and management. J Diabetes Investig.

[10] Bodenheimer T, Wagner EH, Grumbach K (2002). Improving primary care for patients with chronic illness. JAMA.

[11] Fedewa MV, Nickerson BS, Esco MR (2019). Associations of body adiposity index, waist circumference, and body mass index in young adults. Clin Nutr.

[12] Liu J, Tse LA, Liu Z, Rangarajan S, Hu B, Yin L (2019). Predictive values of anthropometric measurements for cardiometabolic risk factors and cardiovascular diseases among 44048 Chinese. J Am Heart Assoc.

[13] Keating SE, Parker HM, Hickman IJ, Gomersall SR, Wallen MP, Coombes JS (2017). NAFLD in clinical practice: Can simple blood and anthropometric markers be used to detect change in liver fat measured by ^1^ H-MRS?. Liver Int.

[14] Choi JR, Koh SB, Choi E (2018). Waist-to-height ratio index for predicting incidences of hypertension: the ARIRANG study. BMC Public Health.

[15] Hou X, Chen S, Hu G, Chen P, Wu J, Ma X (2019). Stronger associations of waist circumference and waist-to-height ratio with diabetes than BMI in Chinese adults. Diabetes Res Clin Pract.

[16] Shen S, Lu Y, Qi H, Li F, Shen Z, Wu L (2017). Waist-to-height ratio is an effective indicator for comprehensive cardiovascular health. Sci Rep.

[17] Ejtahed HS, Kelishadi R, Qorbani M, Motlagh ME, Hasani-Ranjbar S, Angoorani P (2019). Utility of waist circumference-to-height ratio as a screening tool for generalized and central obesity among Iranian children and adolescents: the CASPIAN-V study. Pediatr Diabetes.

[18] Zeng J, Yang RX, Sun C, Pan Q, Zhang RN, Chen GY (2020). Prevalence, clinical characteristics, risk factors, and indicators for lean Chinese adults with nonalcoholic fatty liver disease. World J Gastroenterol.

[19] Lin MS, Lin TH, Guo SE, Tsai MH, Chiang MS, Huang TJ (2017). Waist-to-height ratio is a useful index for nonalcoholic fatty liver disease in children and adolescents: a secondary data analysis. BMC Public Health.

[20] Okamura T, Hashimoto Y, Hamaguchi M, Obora A, Kojima T, Fukui M (2019). Ectopic fat obesity presents the greatest risk for incident type 2 diabetes: a population-based longitudinal study. Int J Obes Lond.

[21] Choi JH, Sohn W, Cho YK (2020). The effect of moderate alcohol drinking in nonalcoholic fatty liver disease. Clin Mol Hepatol.

[22] Hamaguchi M, Kojima T, Itoh Y, Harano Y, Fujii K, Nakajima T, et al. The severity of ultrasonographic findings in nonalcoholic fatty liver disease reflects the metabolic syndrome and visceral fat accumulation. Am J Gastroenterol. 2007;102:2708–15. 10.1111/j.1572-0241.2007.01526.x 17894848

[23] Fitchett EJA, Seale AC, Vergnano S, Sharland M, Heath PT, Saha SK (2016). Strengthening the Reporting of Observational Studies in Epidemiology for Newborn Infection (STROBE-NI): an extension of the STROBE statement for neonatal infection research. Lancet Infect Dis.

[24] Vandenbroucke JP, von Elm E, Altman DG, Gøtzsche PC, Mulrow CD, Pocock SJ (2014). Strengthening the reporting of observational studies in epidemiology (STROBE): explanation and elaboration. Int J Surg.

[25] Wax Y (1992). Collinearity diagnosis for a relative risk regression analysis: an application to assessment of diet-cancer relationship in epidemiological studies. Stat Med.

[26] Tomic D, Kemp WW, Roberts SK (2018). Nonalcoholic fatty liver disease: current concepts, epidemiology and management strategies. Eur J Gastroenterol Hepatol.

[27] Fan JG, Kim SU, Wong VW (2017). New trends on obesity and NAFLD in Asia. J Hepatol.

[28] Zou Y, Yu M, Sheng G (2020). Association between fasting plasma glucose and nonalcoholic fatty liver disease in a nonobese Chinese population with normal blood lipid levels: a prospective cohort study. Lipids Health Dis.

[29] Motamed N, Rabiee B, Hemasi GR, Ajdarkosh H, Khonsari MR, Maadi M (2016). Body roundness index and waist-to-height ratio are strongly associated with non-alcoholic fatty liver disease: a population-based study. Hepat Mon.

[30] Özhan B, Ersoy B, Özkol M, Kiremitci S, Ergin A (2016). Waist to height ratio: a simple screening tool for nonalcoholic fatty liver disease in obese children. Turk J Pediatr.

[31] Zheng RD, Chen ZR, Chen JN, Lu YH, Chen J (2012). Role of body mass index, waist-to-height and waist-to-hip ratio in prediction of nonalcoholic fatty liver disease. Gastroenterol Res Pract.

[32] Cho WK, Kim H, Lee HY, Han KD, Jeon YJ, Jung IA (2015). Insulin resistance of normal weight central obese adolescents in korea stratified by waist to height ratio: results from the Korea National Health and Nutrition Examination Surveys 2008–2010. Int J Endocrinol.

[33] Caminiti C, Armeno M, Mazza CS (2016). Waist-to-height ratio as a marker of low-grade inflammation in obese children and adolescents. J Pediatr Endocrinol Metab.

[34] Tilg H, Moschen AR (2010). Evolution of inflammation in nonalcoholic fatty liver disease: the multiple parallel hits hypothesis. Hepatology.

[35] Day CP, James OF (1998). Steatohepatitis: a tale of two "hits"?. Gastroenterology.

[36] Yilmaz Y (2012). Review article: is non-alcoholic fatty liver disease a spectrum, or are steatosis and non-alcoholic steatohepatitis distinct conditions?. Aliment Pharmacol Ther.

[37] Bessone F, Razori MV, Roma MG (2019). Molecular pathways of nonalcoholic fatty liver disease development and progression. Cell Mol Life Sci.

[38] Chen Z, Yu R, Xiong Y, Du F, Zhu S (2017). A vicious circle between insulin resistance and inflammation in nonalcoholic fatty liver disease. Lipids Health Dis.

[39] Castera L, Friedrich-Rust M, Loomba R (2019). Noninvasive assessment of liver disease in patients with nonalcoholic fatty liver disease. Gastroenterology.

