# Supplementary material for: Waist-to-height ratio and non-alcoholic fatty liver disease in adults
Source: BMC Gastroenterol. 2021 May 25;21:239. doi: 10.1186/s12876-021-01824-3 (PMC8146664; doi:10.1186/s12876-021-01824-3)
Supplement: Supplementary file 1 — Additional file 1. Table S1 Collinearity diagnostics steps. [file 12876_2021_1824_MOESM1_ESM.docx]

Table S1 Collinearity diagnostics steps.

|  | Step 1 | Step 2 | Step 3 | Step 4 |
| --- | --- | --- | --- | --- |
| WHtR | 956.6 | 4.7 | 4.7 | 4.7 |
| Sex | 3.3 | 3.3 | 3.3 | 3.2 |
| Age | 1.4 | 1.4 | 1.4 | 1.4 |
| BMI | 229.2 | 99.2 | 4.9 | 4.9 |
| WC | 1202.4 | NA | NA | NA |
| ALT | 4.1 | 4.1 | 4.1 | 4 |
| AST | 3.3 | 3.3 | 3.3 | 3.3 |
| Weight | 426.9 | 169.3 | NA | NA |
| Habit of exercise | 1 | 1 | 1 | 1 |
| GGT | 1.4 | 1.4 | 1.4 | 1.4 |
| HDL | 1.8 | 1.8 | 1.8 | 1.8 |
| TC | 1.5 | 1.5 | 1.5 | 1.5 |
| HbA1c | 1.2 | 1.2 | 1.2 | 1.2 |
| Smoking status | 1.4 | 1.4 | 1.4 | 1.4 |
| FPG | 1.5 | 1.5 | 1.5 | 1.5 |
| SBP | 5.5 | 5.5 | 5.5 | 1.4 |
| DBP | 5.6 | 5.6 | 5.6 | NA |
| TG | 1.7 | 1.7 | 1.7 | 1.7 |
| Height | 90.8 | 51.6 | 2.5 | 2.5 |
| Drinking status | 1.1 | 1.1 | 1.1 | 1.1 |

VIF = 1/(1-R^2^).
